# The rs696880 Polymorphism in the Nogo-A Receptor Gene (*RTN4R*) Is Associated With Susceptibility to Sporadic Amyotrophic Lateral Sclerosis in the Chinese Population

**DOI:** 10.3389/fnagi.2018.00108

**Published:** 2018-04-12

**Authors:** Lianping Xu, Jiao Li, Danyang Tian, Lu Chen, Lu Tang, Dongsheng Fan

**Affiliations:** ^1^Department of Neurology, Peking University Third Hospital Beijing, China; ^2^Department of Neurology, Beijing Tiantan Hospital, Capital Medical University Beijing, China; ^3^Key Laboratory for Neuroscience, Ministry of Education/National Health & Family Planning Commission, Peking University Beijing, China

**Keywords:** Nogo-A receptor, reticulon 4 receptor gene (*RTN4R*), single-nucleotide polymorphisms, amyotrophic lateral sclerosis, Chinese population

## Abstract

Single-nucleotide polymorphisms (SNPs) in the Nogo-A receptor gene (*RTN4R*) have been associated with increased risk for sporadic amyotrophic lateral sclerosis (SALS) in the French population. In the present study, we investigated the associations between *RTN4R* tag SNPs and SALS in a large Chinese population. Four tag SNPs (rs854971, rs887765, rs696880 and rs1567871) in the *RTN4R* gene with an *r*^2^ threshold of 0.8 and a minor allele frequency (MAF) greater than 0.2% were selected based on Chinese population data from HapMap. A total of 499 SALS patients and 503 healthy controls were genotyped for the SNPs by SNaPshot technology. Haplotype analysis of the four SNPs was performed using the SHEsis software platform. The results showed a significant association between the rs696880 risk allele (A) and SALS in the Han Chinese population (*P* = 0.009, odds ratio (OR) = 1.266 [1.06–1.51]). The allele and genotype frequencies of rs854971, rs887765 and rs1567871 were not associated with SALS. The distribution of the GAAT haplotype was different between the case and control groups (*P* = 0.008, OR = 1.289 [1.066–1.558]). In conclusion, our study showed an association between the *RTN4R* SNP rs696880 and the risk of SALS in the Han Chinese population, with the A allele increasing risk.

## Introduction

Amyotrophic lateral sclerosis (ALS) is a neurodegenerative disease involving brain and spinal cord motor neurons (Wijesekera and Leigh, 2009). Although the incidence of ALS is low, the disease is fatal and has no effective treatment. The disease is considered to result from the interaction of genetics, environment and aging. Gene mutations are a well-known and significant cause of ALS. A twin study estimated that the effect of heritability on ALS is 0.61 and that of an unshared environment is 0.39 (Al-Chalabi et al., 2010). Therefore, genes play a major role in ALS. At least 25 causative genes have been described in ALS, including *SOD1*, *DCTN1*, *ANG*, *VAPB*, *FUS*, *TARDBP*, *UBQLN2*, *PFN1*, *HNRNPA1*, *C9orf72*, *SQSTM1*, *OPTN*, *VCP*, *ATXN2*, *ARHGEF28*, *TUBA4A*, *MATR3*, *CHCHD10*, *TBK1*, *NEKI* and *CCNF* (Brown and Al-Chalabi, 2017; Chia et al., 2018). Among them, *SOD1*, *TARDBP* and C9orf72 are the most common causative genes. Due to differences in genetic backgrounds among different ethnic groups, a difference exists in the frequency of disease-causing gene mutations in ALS patients. Zou et al. (2017) performed a meta-analysis of the mutation frequency of these four common genes and found that in the European population, the most common pathogenic gene was *C9orf72* (familial ALS [FALS] 33.7%, sporadic ALS [SALS] 5.1%), followed by *SOD1* (FALS 14.8%, SALS 1.2%), *TARDBP* (FALS 4.2%, SALS 0.8%) and *FUS* (FALS 2.8%, SALS 0.3%). However, in the Asian population, *SOD1* (FALS 30.0%, SALS 1.5%) was the most common gene, followed by *FUS* (FALS 6.4%, SALS 0.9%), *C9orf72* (FALS 2.3%, SALS 0.3%) and *TARDBP* (FALS 1.5%, SALS 0.2%).

In our previous study, the mutation rates of *SOD1*, *FUS*, *TARDBP* and *C9orf72* in FALS patients were 34.5%, 9.5%, 4.3% and 0%, respectively (Liu R. et al., 2013). The mutation rates of *SOD1*, *FUS*, *TARDBP* and *C9orf72* in SALS patients were 2.2%, 3.0%, 1.1% and 0.3%, respectively (He et al., 2015). The mutation rates of other genes were as follows:* ATXN2*, 1.6% (Liu X. et al., 2013); *ARHGEF28*, 0.56% (Ma et al., 2014); *SQSTM1*, 1.38% (Yang et al., 2015);* OPTN*, 0.78% (Li C. et al., 2015); *TUBA4A*, 0% (Li J. et al., 2015); *MATR3*, 0.19% (Xu et al., 2016); *DCTN1*, 0.39% (Liu et al., 2017); *UBQLIN*, 0.19% (Huang et al., 2017); and *CHCHD1*, 0.23% (Shen et al., 2017).

Approximately 10% of ALS cases are FALS, while approximately 90% of cases are SALS. The cause of SALS remains unknown. SALS may result from the interaction between susceptible genes and the environment; therefore, identifying SALS susceptibility genes is as important as identifying pathogenic genes. Several previous studies have identified different susceptibility genes in various ethnic cohorts, including *DPP6*, *ITPR2*, *UNC13A*, *FGGY*, *ELP3*, *KIFAP3*, *9p21.2*, *ZNF512B*, *TIMA1*, *SCNN1A*, *BDNF*, *C21orf2*, *MOBP*, *SCFD1* and *GPX3-TNIP1* (van Es et al., 2007, 2008, 2009a,b; Cronin et al., 2008; Landers et al., 2009; Simpson et al., 2009; Iida et al., 2011; Cai et al., 2014; Chen et al., 2014; van Rheenen et al., 2016; Xu et al., 2017).

Although many ALS-related genes have been identified, approximately 44% of FALS and 91% of SALS cases in European populations and 59% of FALS and 96% of SALS cases in Asian populations still have no clear pathogenesis (Zou et al., 2017). Therefore, the genetic study of pathogenic genes in Chinese ALS patients is of great significance.

Recently, two non-coding variants (rs701427 A and rs1567871 C) in the Nogo-A receptor gene (*RTN4R*) were reported to confer susceptibility to SALS in the French population (Amy et al., 2015). Moreover, rs701427 showed a significant correlation with reduced gene expression. The study concluded that reduced expression of the Nogo receptor was associated with SALS. In this study, we investigated the associations between *RTN4R* tag single-nucleotide polymorphisms (SNPs) and SALS in a large Chinese population.

## Materials and Methods

### Study Population

This study consisted of 499 SALS patients and 503 healthy control subjects. All participants were of Han Chinese origin. Diagnosis of ALS was performed at Peking University Third Hospital using the E1 Escorial Word Federation criteria for definite or probable ALS (Brooks et al., 2000). This study was carried out in accordance with the recommendations of Peking University Third Hospital ethics committee with written informed consent from all subjects. All subjects gave written informed consent in accordance with the Declaration of Helsinki. The protocol was approved by the Peking University Third Hospital ethics committee.

### SNP Selection

Based on the Chinese population data from the HapMap database (HapMap Data Rel 27 Phase II+III, Feb 2009, on NCBI B36 assembly, dbSNP126), candidate tag SNPs in *RTN4R* with an *r*^2^ threshold of 0.8 and a minor allele frequency (MAF) greater than 0.2% were selected.

### SNP Analysis

Genomic DNA was extracted from leukocytes of venous blood using the phenol-chloroform method. The sense and antisense primers for SNPs are shown in Table 1. Primers were used to amplify the target SNPs, followed by agarose gel electrophoresis and product recovery. The product was primer-extended by one base and terminated. It was detected by an ABI sequencer at Tsingke Biotechnology Co. (Beijing, China) according to the ABI PRISM^®^ SNaPshot™ Multiplex Kit Protocol.

**Table 1:** The primes of the single-nucleotide polymorphisms (SNPs).

SNP	Sense primers	Antisense primers
rs854971	CCCACTTTCCACTCTCACCC	GGAGGAGACCCTGGAGGAAA
rs887765	GGAGAATTTTGGTTGGGCCTAGA	CTGACCCTCTGTGGTGTGGG
rs696880	ATCCTCAGGCCCACAGACAC	GGTCCAGTGACCTCTCACCA
rs1567871	CCCAGAGAAAGGGGTTCCAC	ATGCCCAGTGGAGGACTG

### Statistical Analysis

The chi-square test was used to compare the allele frequencies and genotype differences between the case and control groups. Odds ratios (ORs) and 95% confidence intervals (CIs) were used to assess the risk of genetic polymorphisms. Haplotype analysis was performed with the SHEsis software platform^1^ (Shi and He, 2005; Li et al., 2009). The original *P* value was multiplied by the number of comparisons to obtain a Bonferroni-corrected *P* value. Corrected *P*-values less than 0.05 were considered significant.

### Functional Prediction

The functions of positive loci were analyzed using MATCH software.

## Results

**Figure 1:**
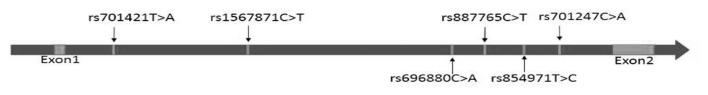
Genomic structure and localization of polymorphisms in the *RTN4R* gene.

A total of four SNPs of *RTN4R* (rs854971, rs887765, rs696880 and rs1567871) were selected for further genotyping. All four SNPs were located in the intron between exon 1 and exon 2 (Figure 1).

This study included 499 SALS patients with a male-to-female ratio of 1.80 and average age of onset of 51.3 ± 11.9 years. The study also included 503 control subjects, with a male-to-female ratio of 1.30 and a mean age of 53.5 ± 12.8 years. No difference was observed between the patients and control subjects in age or sex.

The four polymorphisms (rs854971, rs887765, rs696880 and rs1567871) were in Hardy-Weinberg equilibrium in SALS patients and control subjects.

The rs696880 A allele (*P* = 0.009, OR = 1.266 [1.06–1.51]) and rs1567871 T allele (*P* = 0.045, OR = 1.204 [1.00–1.44]) were associated with SALS in terms of allele and genotype frequencies (Table 2). Since four SNPs were included in the study, the Bonferroni-corrected *P* value of the allele or genotype comparison was the original *P* value multiplied by 4. Hence, only the rs696880 allele distributions was significantly correlated with SALS after Bonferroni correction. The allele and genotype frequencies of rs854971 (*P* = 0.053, OR = 0.836 [0.69–1.01]) and rs887765 (*P* = 0.112, OR = 1.161 [0.96–1.39]]) were not associated with SALS.

**Table 2:** Allele and genotype frequencies of the four SNPs within *RTN4R*.

SNP	Samples	*n*	Allelic (frequency)		*P*^a^(χ^2^)	OR (95%CI)	Genotype (frequency)			*P*^b^(χ^2^)
			A	G			AA	AG	GG	
rs854971	Cases	499	357 (0.360)	641 (0.642)	0.053	0.836 [0.69~1.01]	66 (0.132)	225 (0.450)	208 (0.416)	0.162
	controls	503	402 (0.400)	604 (0.600)	(3.736)		89 (0.177)	224 (0.445)	190 (0.378)	(3.631)
			A	G			AA	AG	GG	
rs887765	Cases	499	357 (0.357)	641 (0.642)	0.112	1.161 [0.96~1.39]	59 (0.123)	239 (0.484)	201 (0.393)	0.067
	controls	503	326 (0.324)	680 (0.676)	(2.527)		57 (0.113)	212 (0.421)	234 (0.465)	(5.413)
			A	G			AA	AG	GG	
rs696880	Cases	499	489 (0.490)	509 (0.510)	0.009	1.266 [1.06~1.51]	117 (0.234)	255 (0.511)	127 (0.254)	0.027
	controls	503	434 (0.431)	572 (0.569)	(6.917)		95 (0.189)	244 (0.485)	164 (0.326)	(7.214)
			C	T			CC	CT	TT	
rs1567871	Cases	499	574 (0.575)	424 (0.425)	0.045	1.204 [1.00~1.44]	158 (0.320)	258 (0.506)	83 (0.174)	0.018
	controls	503	585 (0.626)	359 (0.374)	(4.002)		202 (0.402)	225 (0.448)	75 (0.149)	(8.029)

*^a^d.f. = 1; ^b^d.f. = 2. d.f, degree of freedom. P value in the table is the original P value*.

Using the method of increasing sensitivity with the lowest false-negative rate, MATCH software predicted that if allele A replaced G in rs696880, one more cooperates with myogenic proteins 1 (COMP1) transcription factor binding site would be expected.

Haplotype frequencies were calculated using the SHEsis software platform. According to the sequence of rs854971-rs887765-rs696880-rs1567871, the AGGC, GAAT, GGAC, GGAT and GGGC haplotypes, with the smallest haplotype frequency >0.03, were included in the study as common haplotypes. The GAAT haplotype and non-GGAT haplotypes differed between the case group and the control group (*P* = 0.008). Because the GAAT haplotype was involved in five multiple comparisons, *P* = 0.008 × *5* = 0.04 after Bonferroni correction; therefore, this difference was still significant (Table 3).

**Table 3:** Haplotype frequencies of the tag SNPs within *RTN4R*.

Haplotype^a^	Case (freq)^b^	Control (freq)^b^	χ^2^	Pearson’s P	Odds ratio[95%]
AGGC	349.47 (0.345)	374.75 (0.373)	3.196	0.073	0.845 [0.703~1.016]
GAAT	353.95 (0.350)	288.21 (0.287)	6.880	0.008	1.289 [1.066~1.558]
GGAC	57.93 (0.057)	47.34 (0.050)	0.766	0.381	1.193 [0.804~1.770]
GGAT	70.62 (0.070)	74.84 (0.075)	0.343	0.558	0.904 [0.645~1.268]
GGGC	156.38 (0.153)	168.84 (0.168)	1.226	0.268	0.874 [0.689~1.109]

*^a^The sequence of the SNPs (rs854971-rs887765-rs696880-rs1567871). ^b^The frequencies of haplotype were estimated using SHEsis platform software. Common haplotype included in this study were selected with minor frequency greater than 0.03*.

## Discussion

The tag SNPs in the *RTN4R* gene are different between the Han Chinese population and the French population. Four SNPs, rs854971, rs887765, rs696880 and rs1567871, were included in our study. Three SNPs, rs701427, rs701421 and rs1567871, were included in the French study (Amy et al., 2015). The MAFs of rs701427 and rs701421 were both lower than 0.2% in the Han Chinese population; therefore, these two SNPs were not included in our study. Only the rs1567871 SNP was included in both studies. In Amy et al.’s (2015) study, the rs1567871 C allele was associated with SALS in the French population. However, the rs1567871 C allele was not a risk allele for SALS in the Chinese population. This difference may be attributable to ethnic factors. The *C9orf72* gene frequency was high in European and American ALS patients (FALS 33.7%, SALS 5.1%) but low in Asian ALS patients (FALS 2.3%, SALS 0.3%; Zou et al., 2017). The frequency of *OPTN* mutations was high in Japanese and Chinese populations (FALS 3.3%–3.8%, SALS 0.23%–1%), whereas these mutations occurred infrequently in Caucasian patients (Li C. et al., 2015).

Nogo-A is a myelin growth inhibitor protein that exerts an inhibitory effect by binding to the Nogo-A receptor (NgR1). NgR1 is a glycosylphosphatidylinositol (GPI)-linker that is rich in leucine-rich repeats (LRRs) and is encoded by the endoplasmic reticulum-4 receptor (*RTN4R*) gene. NgR1 forms a receptor complex with Lingo-1, p75NTR and Troy, binding to the growth inhibitory protein Nogo-A, oligodendrocyte myelin-associated glycoprotein (OMG), and myelin-binding glycoprotein myelin-associated glycoprotein (MAG; Schmandke et al., 2014).

Rho-A, a GTP-binding protein, can activate the signal transduction that regulates NgR1 in neurons, leading to growth inhibition and congenital atrophy (Fournier et al., 2002; GrandPré et al., 2002). Motor neuron growth inhibition leads to denervation of muscles and is an important pathological mechanism of ALS (Jokic et al., 2006; Steele and Yi, 2006). However, the NgR1 receptor may also regulate the complex role of maintaining motor neuron survival in ALS patients by blocking the p75NTR-mediated neuronal death induced by nerve growth factor (NGF; Dupuis et al., 2008). This shows that the Nogo-A/NgR1 signaling pathway and the complex role of NgR1 in ALS are closely related (Teng and Tang, 2008; Schmandke et al., 2014). Amy et al. (2015) concluded that expression of the Nogo receptor is positively associated with SALS.

Our study suggests that the rs696880 A allele in the *RTN4R* gene increases the risk of SALS in the Han Chinese population. However, the mechanism is not clear. The rs696880 SNP, located in an enhancer element of the *RTN4R* gene, may regulate *RTN4R* gene transcription by changing COMP1 transcription factor binding sites. As concluded by Amy et al. (2015), the A allele of rs696880 may reduce transcription of the RTN4R gene, but this requires further functional verification.

The above findings demonstrate that Nogo-A/NgR1 contributes to the neuropathology of ALS. Variation at the NgR locus is associated with schizophrenia (Hsu et al., 2007; Budel et al., 2008; Voineskos, 2009; Jitoku et al., 2011; Willi and Schwab, 2013). Hence, our data provide additional evidence for the view that a genetic overlap exists between ALS and schizophrenia (Byrne et al., 2013; Fahey et al., 2014).

To the best of our knowledge, this is the first analysis of *RTN4R* tag SNPs in Han Chinese patients with SALS. This case-control study may provide an association between the Nogo-A receptor gene SNP rs696880 and the risk of SALS in the Han Chinese population, with the A allele increasing risk. However, the results still need to be verified in a larger study. Larger studies in different ethnicities are also needed to confirm and extend our findings.

## Author Contributions

DF conceived this study and provided financial support and was responsible for project management. LX, JL and DT performed the experiments and analyzed the data. LC and LT conducted data management and undertook data checking. LX and DF wrote the manuscript.

## Conflict of Interest Statement

The authors declare that the research was conducted in the absence of any commercial or financial relationships that could be construed as a potential conflict of interest.

## Footnotes

^1^http://analysis.bio-x.cn
